# Syndrome of Inappropriate Antidiuretic Hormone Secretion (SIADH) Triggered by Heat Stroke: A Case Report

**DOI:** 10.7759/cureus.85304

**Published:** 2025-06-03

**Authors:** Kenko Aoki, Keisuke Suzuki, Takuya Shimada, Rei Ogawa, Kenji Dohi

**Affiliations:** 1 Department of Plastic, Reconstructive and Aesthetic Surgery, Nippon Medical School, Tokyo, JPN; 2 Department of Emergency, Critical and Disaster Medicine, Showa University School of Medicine, Tokyo, JPN

**Keywords:** cns complications, heat stroke, oxidative stress, syndrome of inappropriate secretion of antidiuretic hormone (siadh), treatment of hyponatremia

## Abstract

We present the first documented case of the syndrome of inappropriate antidiuretic hormone secretion (SIADH) triggered by heat stroke in a man in his mid-50s with no medical history. After experiencing acute symptoms during a heatwave, he was hospitalized. The absence of dehydration and the presence of hyponatremia, along with other laboratory findings, fulfilled the diagnostic criteria for SIADH once other causes were excluded. The linkage between heat stroke and SIADH is thought to involve neuroinflammation and oxidative stress in the hypothalamus, which impacts antidiuretic hormone (ADH) production and secretion. This case highlights critical considerations for emergency and post-acute care in heat-related illnesses, emphasizing the need for vigilant monitoring of SIADH symptoms in patients experiencing heat stroke.

## Introduction

Heat stroke is a systemic condition caused by exposure to high ambient temperatures and relative humidity. Its prevalence is estimated to increase due to recent global warming, making it a serious worldwide social issue [1]. The syndrome of inappropriate antidiuretic hormone secretion (SIADH) accounts for approximately one-third of all cases of hyponatremia and is the most common cause in euvolemic patients. SIADH is a disorder characterized by impaired water excretion due to excessive or inappropriate secretion of antidiuretic hormone (ADH). Unlike other causes of hyponatremia such as hypovolemia or heart failure, SIADH typically presents with normal fluid volume status and inappropriately concentrated urine [2]. Common etiologies of SIADH include medications, malignancies, pulmonary infections, central nervous system (CNS) disorders, severe pain, and nausea. However, not all triggers for SIADH are fully understood. For instance, a study in a general hospital found that idiopathic SIADH was the most common diagnosis (39.8%), followed by pulmonary disorders (34.2%), medications (10%), CNS etiologies (8.4%), and malignancies (7.3%) [3]. No previous reports have documented an association between heat stroke and SIADH. Here, we present the first reported case of heat stroke complicated by SIADH.

This article was previously presented as a meeting abstract at the Society of Critical Care Medicine (SCCM) 2023 conference in January 2023.

## Case presentation

**Figure 1:**
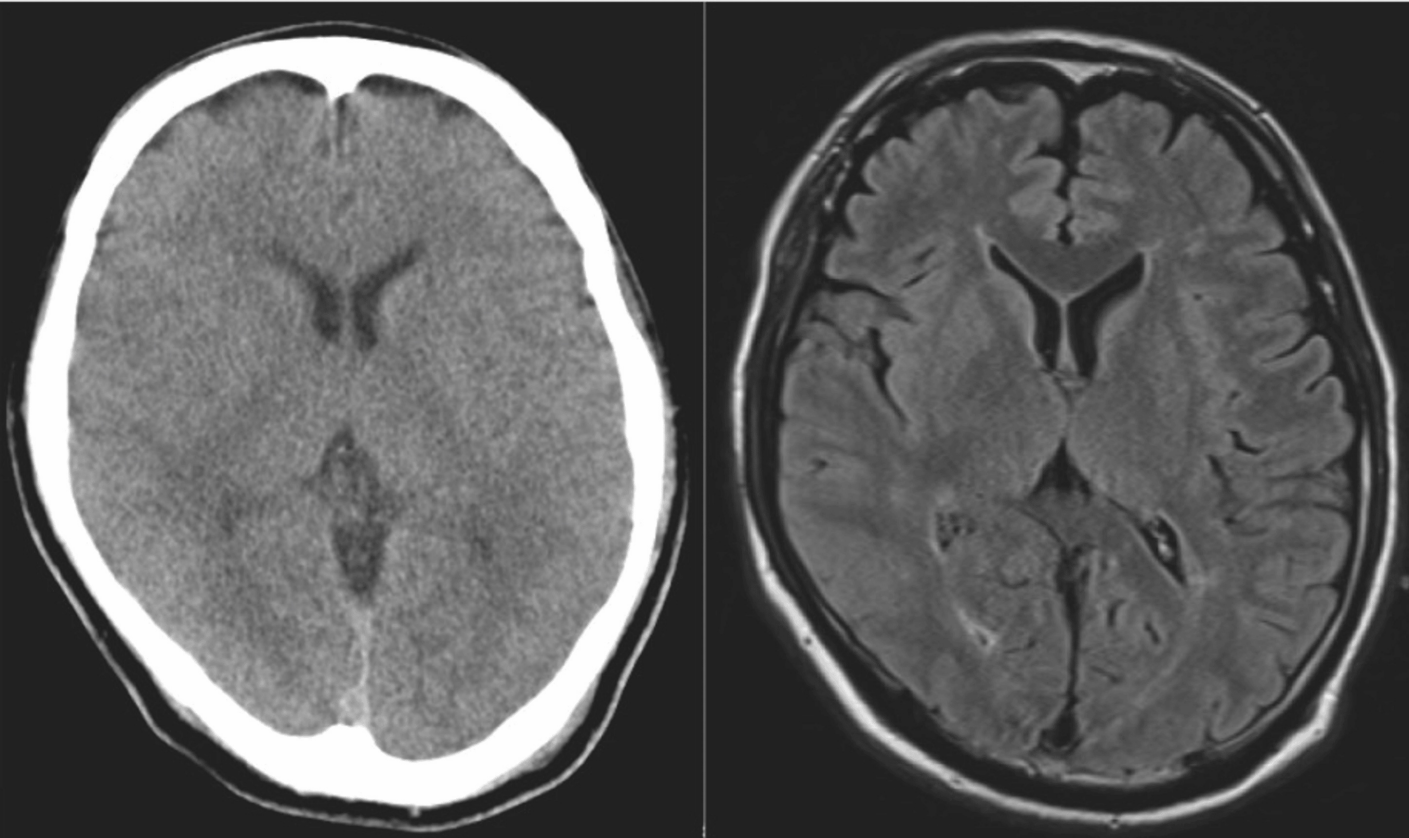
Plain head CT scan and contrast-enhanced T1-weighted MRI A plain head CT scan (left) and contrast-enhanced T1-weighted MRI (right) performed on the patient's arrival at the hospital showed no obvious cranial abnormalities.

**Figure 2:**
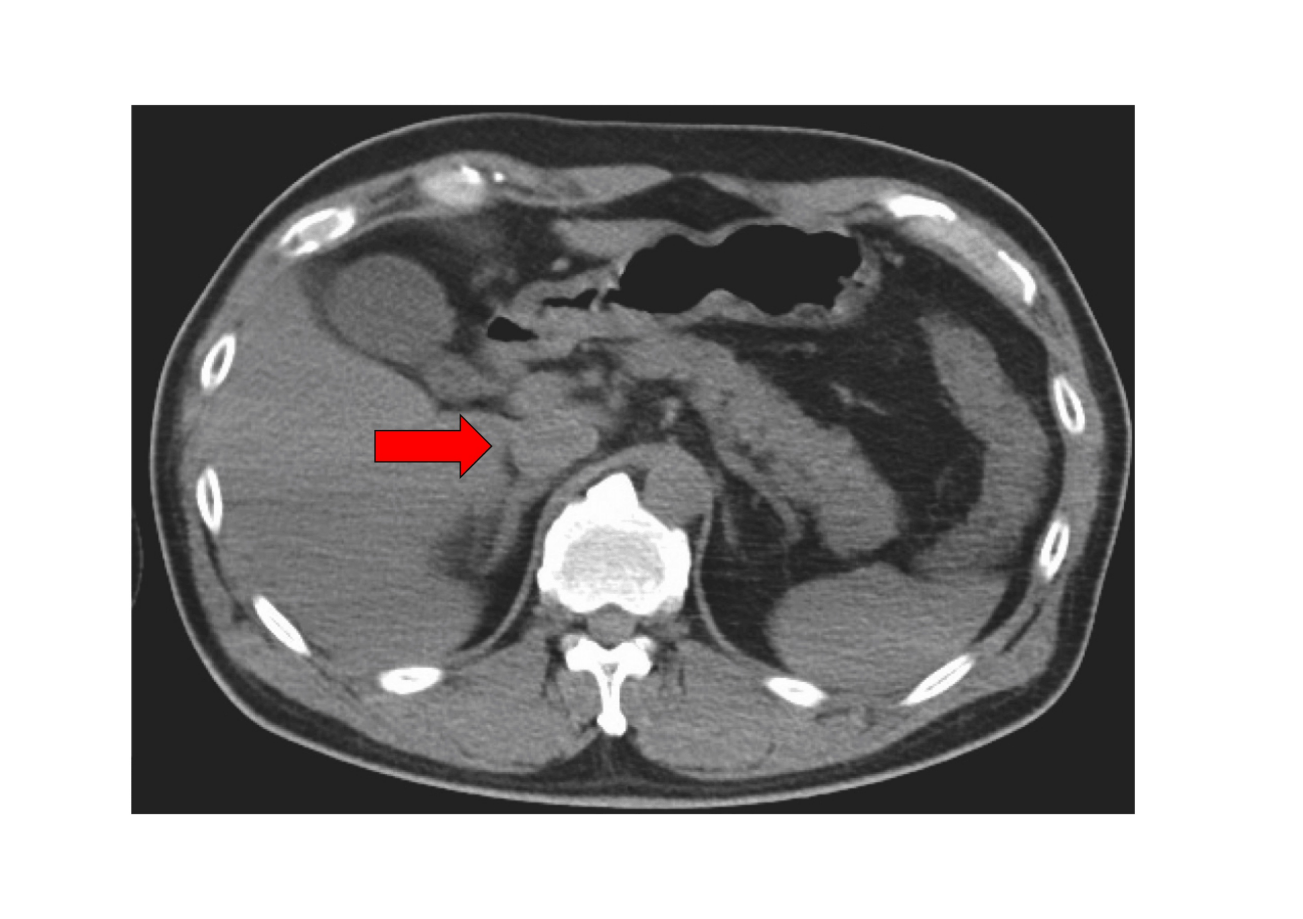
Plain abdominal CT scan A plain abdominal CT scan performed upon the patient's arrival at the hospital showed that the inferior vena cava (red arrow) was neither distended nor collapsed, with no obvious abnormalities detected. These findings suggest that the patient was not significantly dehydrated despite exposure to high temperatures.

**Figure 3:**
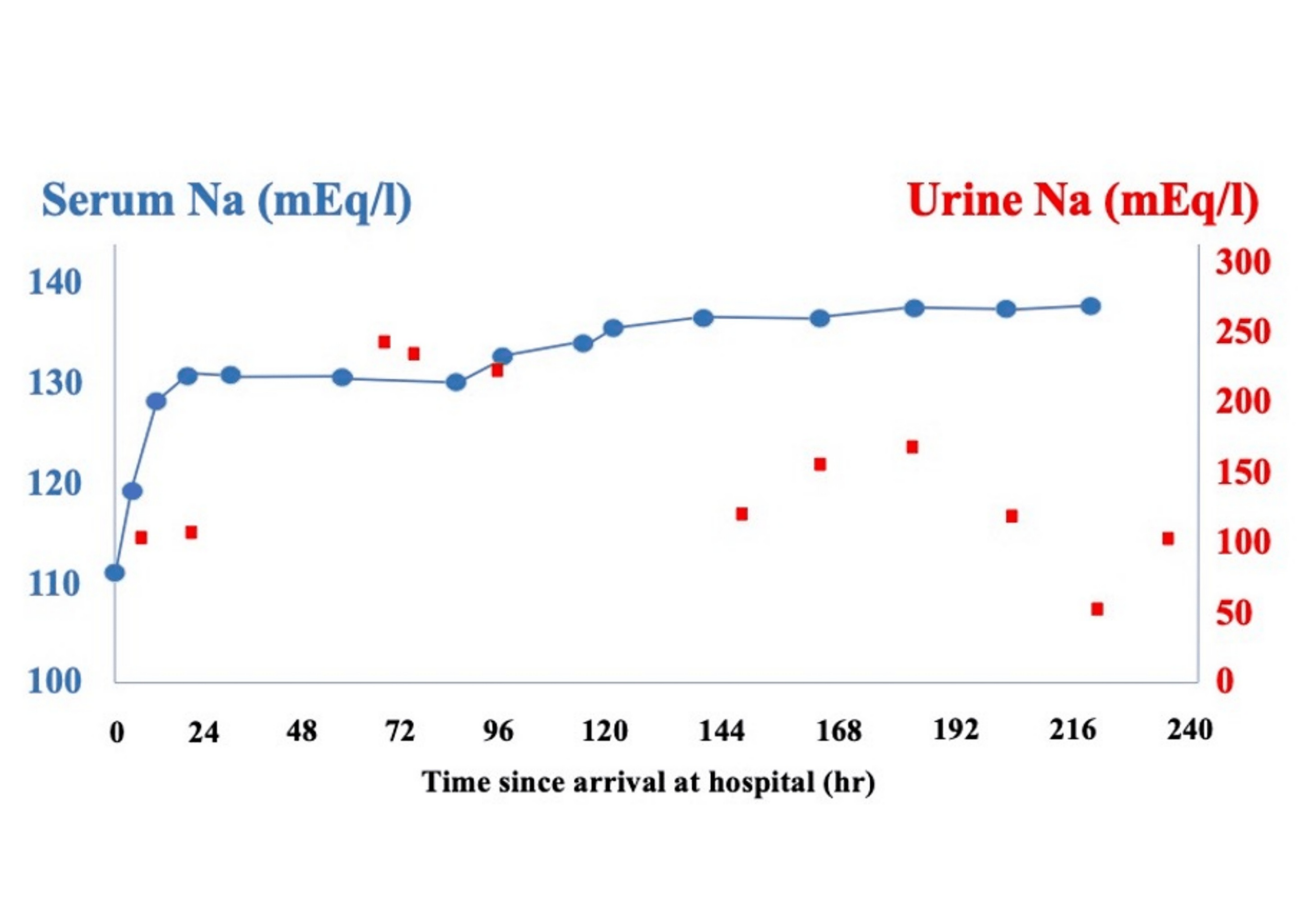
Clinical course The clinical course up to 240 hours after the patient's arrival at the hospital shows serum sodium (left axis) and urine sodium (right axis) levels in milliequivalents per liter (mEq/L). Sodium correction was initiated upon arrival, and the serum sodium level improved to 130 mEq/L within 24 hours of treatment.

A 54-year-old male patient had no history of heat stroke or any endocrine disorder. He was living in a room without air-conditioning equipment during a heat wave with temperatures exceeding 35°C every day. He had been consuming regular meals with no notable abnormalities in dietary intake, and there was no history of excessive fluid consumption or polydipsia. He had felt unwell since the previous day. After breakfast, he remained in the bathroom for several hours, a confined and poorly ventilated space, which resulted in prolonged exposure to extreme heat. When the bathroom door was opened, he was found collapsed on the floor and exhibiting convulsive movements, prompting a call for emergency medical assistance. Neither vomiting nor diarrhea was observed. When he arrived at the hospital, his convulsions had ceased, and his vital signs were as follows: pulse 124 beats/min, blood pressure 129/77 mmHg, respiratory rate 30 breaths/min, SpO₂ 97% on room air, and rectal temperature 42.0℃. His level of consciousness was decreased to E1V1M4/GCS, but there was no apparent paralysis. Blood tests showed a mildly elevated inflammatory response and hyponatremia, with a sodium level of 110.4 mEq/L (Table 1). CT and MRI scans showed no obvious intracranial lesions (Figure 1). The patient's loss of consciousness was diagnosed as due to heat stroke and hyponatremia. Active cooling interventions were started. These included the surface cooling of ice packs, reduction of ambient temperature in the emergency room, and intravenous infusion of chilled crystalloid fluids. The patient was admitted to the ICU. Physical examination revealed no dehydration. The abdominal CT scan showed no abdominal fluid and the inferior vena cava was not collapsed (Figure 2). Laboratory tests showed a plasma osmolality of 244 mOsm/kg, urine osmolality of 423 mOsm/kg, urinary sodium concentration of 99 mEq/L, ADH level of 43.1 pg/mL, and cortisol level of 46.8 μg/dL. These results supported the diagnosis of SIADH. The values of BUN and Cre were 16.5 mg/dL and 1.55 mg/dL, respectively, findings that were not suggestive of a dehydrated state. Na loading was administered. On the first day, we administered 4000 mL of crystalloid infusion and 500 mL of 3% NaCl infusion. This led to a recovery of serum sodium to 130 mEq/L. On the second day, the patient received 1000 mL of crystalloid solution and 1000 mL of 3% NaCl. The patient’s serum sodium level improved to 136.1 mEq/L by the seventh day (Figure 3). By the 11th day, his level of consciousness had improved to E4V4M6 on the Glasgow Coma Scale (GCS), and he was transferred to a rehabilitation hospital on the 13th day. At a follow-up visit one year after discharge, a complete resolution of SIADH was confirmed. Cognitive function, including memory, was within normal limits, with no evidence of neurocognitive impairment or other neurological sequelae.

**Table 1:** Laboratory test results on arrival at the hospital The laboratory test was conducted upon the patient's arrival at the hospital. It showed plasma osmolality of 244 mOsm/kg, urine osmolality of 423 mOsm/kg, urinary Na concentration of 99 mEq/L, ADH of 43.1 pg/mL, and cortisol of 46.8 μg/dL, consistent with a diagnosis of SIADH. The values of BUN and Cre were 16.5 mg/dL and 1.55 mg/dL, respectively, which did not indicate that the patient was in a dehydration status.

Category	Parameter	Result	Reference Range	Unit
Blood Count	White Blood Cell (WBC)	13800	3,500-9,000	/μL
	Hemoglobin (Hb)	14.5	13.5-17.5	g/dL
	Hematocrit (Ht)	38.8	40-50	%
	Platelets (Plt)	256	150-350	×10^3^/μL
Biochemistry	Sodium (Na)	110	135-145	mEq/L
	Potassium (K)	4	3.5-5.0	mEq/L
	Chloride (Cl)	83	98-106	mEq/L
	Calcium (Ca)	6.1	8.5-10.5	mg/dL
	Blood Urea Nitrogen (BUN)	16.5	7-20	mg/dL
	Creatinine (Cre)	1.55	0.6-1.2	mg/dL
	Uric Acid (UA)	5	3.6-7.0	mg/dl
	AST	74	10-40	U/L
	ALT	83	7-56	U/L
	Creatine Kinase (CK)	984	30-200	U/L
	CK-MB	2.5	0-5	ng/mL
	Myoglobin (Mb)	3996	0-85	ng/mL
	C-Reactive Protein (CRP)	0.07	<0.3	mg/dL
Endocrine	Antidiuretic Hormone (ADH)	43.1	0.3-3.5	pg/mL
	ACTH	141.2	7.7-63.1	pg/mL
	Cortisol	46.8	5.1-17	μg/dL
	Thyroid Stimulating Hormone (TSH)	0.33	0.4-4.0	μIU/mL
	Free T4 (FT4)	1.03	0.8-1.8	ng/dL
	Free T3 (FT3)	2.15	2.3-4.0	pg/mL
Urine	Urine BUN	286	-	
	Urine Creatinine	42.3	20-275	mg/dL
	Urine Sodium (Na)	99	40-220	mEq/L
	Urine Potassium (K)	19.8	3-20	mEq/L
	Urine Chloride (Cl)	104	25-40	mEq/L
	Urine Osmolality (OSM)	423	300-900	mOsm/kg

## Discussion

In our patient, the diagnosis of SIADH was established based on laboratory findings: low plasma osmolality, high urinary osmolality, elevated urinary sodium concentration, an increased ADH level, and an elevated cortisol level, all in the absence of dehydration or other potential causes such as adrenal or thyroid dysfunction [2]. The primary trigger was thought to be heat stroke, which is not a well-documented cause of SIADH. This case suggests that heat stroke-induced CNS damage may act as a trigger for SIADH.

The diagnosis of heat stroke is mainly clinical and is primarily based on the triad of hyperthermia (usually >40.5℃) and neurologic abnormalities [1]. This condition results from exposure to high environmental temperatures or strenuous physical activity and can lead to progressive multi-organ failure and cerebellar damage [4].

The mechanism linking heat stroke to SIADH is not yet fully understood. However, reports have indicated that heat stroke can cause cerebral edema and neuroinflammation [5,6]. Animal studies have revealed that heat stress induces neuroinflammation and oxidative stress in the hypothalamus, a region critical for ADH production and secretion [7,8]. Additionally, proteomic analyses in animal models have demonstrated differential expression of endocrine-related proteins in the hypothalamus and pituitary under heat stress [9,10]. These findings suggest a pathophysiological process similar to that observed in neuropathies caused by trauma or inflammation, which are the causes of SIADH [6,11]. 

In this case, severe hyponatremia and impaired consciousness were likely due to SIADH, triggered by CNS damage from heat stroke. Prolonged impaired consciousness was observed even after serum sodium levels normalized, further supporting the involvement of heat stroke-induced CNS dysfunction. Given the absence of other plausible causes of SIADH, our findings strengthen the hypothesis that heat stroke can induce SIADH.

Managing SIADH in patients with heat stroke presents significant clinical challenges. The standard treatment for SIADH involves fluid restriction; however, this approach complicates cases where heat stroke may induce SIADH, often presenting with hyponatremia and altered consciousness. Distinguishing the underlying cause of altered consciousness is challenging, as both heat stroke and hyponatremia can independently impair cognitive function.

In this case, the patient was hydrated with 4000 mL of crystalloid solution and 500 ml of 3% NaCl infusion on the first day, resulting in an increase in serum sodium to 130 mEq/L. Following a noted decrease in serum sodium without additional sodium intake on the second day, the patient received 1000 mL of crystalloid solution and 1000 mL of 3% NaCl. Initially, there was only a minor improvement in serum sodium levels, and the patient's impaired consciousness persisted, leading us to aggressively correct the electrolyte imbalance. This resulted in an appreciable increase in serum sodium. Reflecting on this, we understand that although acute hyponatremia has a lower risk of central pontine myelinolysis from rapid correction than chronic cases, the rapid increase still presented significant risks. A more cautious approach, limiting correction to no more than 12 mEq/L of serum sodium within the first 24 hours, would have been safer [12]. More frequent monitoring of serum sodium levels during the correction phase may have allowed for earlier intervention in response to an excessively rapid correction rate. 

Both CSWS (Cerebral Salt-Wasting Syndrome) and SIADH are causes of hyponatremia with similar clinical presentations. In the treatment of CSWS, replenishing sodium and fluids can improve sodium levels. Due to the relatively rapid normalization of serum sodium with sodium and fluid supplementation, symptomatic recovery is generally quick if the treatment is effective. However, in SIADH, if fluid restriction and sodium management are not continued, there is a tendency for sodium levels to decrease again. This clinical response confirmed the diagnosis of SIADH. Several other features help distinguish between them. SIADH is characterized by a normal fluid status, whereas CSWS presents with hypovolemia. In addition, a fractional excretion of uric acid (FEurate) after correction of hyponatremia greater than 11% suggests CSWS, whereas a value below 11% supports a diagnosis of SIADH [13]. In this case, the patient was euvolemic at presentation. On day five of admission, when the serum sodium level had recovered to 134.5 mg/dL, the FEurate was calculated to be 7.1%. These findings are inconsistent with CSWS and support a diagnosis of SIADH.

As heat stroke can cause CNS damage, SIADH triggered by heat stroke should be categorized under CNS etiologies. There is a well-established association between CNS etiologies and severe cases of SIADH [2]. Therefore, patients diagnosed with SIADH in the context of heat stroke require active treatment and close monitoring. Since this report describes only a single case, further prospective studies are needed to investigate the frequency and underlying mechanisms of SIADH in patients with heat stroke.

## Conclusions

Heat stroke can cause CNS damage, potentially triggering the development of SIADH. Given its implications on cognitive function, managing SIADH following heat stroke requires meticulous regulation of sodium and fluid levels. This case analysis underscores the necessity of classifying SIADH associated with heat stroke as a consequence of CNS damage, requiring immediate and vigorous treatment as well as comprehensive monitoring.
